# Involvement of miRNA203 in the proliferation of epidermal stem cells during the process of DM chronic wound healing through Wnt signal pathways

**DOI:** 10.1186/s13287-020-01829-x

**Published:** 2020-08-12

**Authors:** Jian Liu, Bin Shu, Ziheng Zhou, Yingbin Xu, Yiling Liu, Peng Wang, Kun Xiong, Julin Xie

**Affiliations:** 1 Department of Burn Surgery, First Affiliated Hospital of Sun Yat-Sen University, No. 58, 2nd Zhongshan Road, Yuexiu District, Guangzhou City, 510080 Guangdong Province People’s Republic of China; 2 Department of Anatomy and Neurobiology, School of Basic Medical Sciences, Central South University, Changsha, 410013 Hunan People’s Republic of China

**Keywords:** Epidermal stem cells, Wound healing, Diabetes mellitus chronic wound, miR-203, Signaling pathway

## Abstract

**Background:**

The biological role of miR-203 and the underlying mechanisms on the proliferation of epidermal stem cells (ESCs) have not yet been reported during the progression of chronic wound healing in diabetes mellitus. Our previous studies have observed that the expression of miR-203 showed a marked upregulation and ESC proliferation capacity was impaired in diabetes mellitus skin wounds in rats.

**Methods:**

Wound models were established in normal rats and rats with type 2 diabetes. Expression level of miR-203 and the alteration of ESCs’ number and function were detected. ESCs were isolated from the back skin of fetal rats to assess the effects of glucose in vitro. An antagomir to miR-203 was used to assess its effect on ESCs. Using microarray analysis, we further identified potential target genes and signaling pathways of miR-203.

**Results:**

We found that high glucose significantly upregulated the expression of miR-203 and subsequently reduced the number of ESCs and impaired their proliferation capacity. Meanwhile, over-expression of miR-203 reduced the ESCs’ numbers and impaired the proliferation capacity via downregulation of the Notch and Wnt signaling pathways. Conversely, inhibition of miR-203 enhanced the proliferation capacity. Additionally, silencing miR-203 in skin of rats with type 2 diabetes accelerated wound healing and improved healing quality via the upregulation of the Notch and Wnt signaling pathways. Finally, over-expression of miR-203 downregulated genes ROCK2, MAPK8, MAPK9, and PRKCA.

**Conclusion:**

Our findings demonstrated that induced expression of miR-203 by high glucose in type 2 diabetic rats decreased the number of ESCs and impaired ESC proliferation capacity via downregulating genes related to Notch and Wnt signaling pathways, resulting in a delayed wound healing.

## Background

With the progression of diabetes mellitus (DM), chronic complications such as diabetic neuropathy or microangiopathy often lead to refractory wounds in lower extremities, which might result in amputation or even worse [1]. To date, emerging evidence has indicated that diabetic wounds are not only a result of hyperglycemia, advanced glycation end-products (AGEs), microangiopathy, and aberrant expression of matrix metalloproteinases (MMPs), but also a consequence of proliferative dysfunction of epidermal stem cells (ESCs) [2, 3]. Studies have shown that the ESCs from diabetic patients were decreased in proliferation, less in quantity, dysregulated in adhesive activity, and abnormal in distribution [4]. These changes can occur prior to the development of wounds and may participate in impairment of wound healing in diabetic patients [5–8]. However, diabetes induced these biological anomalies of ESCs through a hitherto unknown mechanism.

The ESCs are equipped with the capacity to proliferate and potential to differentiate into multiple kinds of cells [9]. In wounds, the ESCs participate in numerous biological processes such as focal adhesion, extracellular matrix synthesis, and cell migration via proliferation and differentiation in different stages of wound healing [9]. The “stem cell niche,” the micro-environment which the stem cells located, exerts a profound impact on the regulation of proliferation and differentiation of the ESCs, and both Wnt and Notch signaling pathways play a key role in the specific mechanism [10, 11]. In wound healing process, the Wnt/β-catenin pathway is activated to facilitate the coverage of stratified epithelial on wounds. Respectively, the canonical Notch pathway is also involved in the regulation of proliferation/differentiation of stem cells [12]. However, the influence of the Wnt and Notch signaling pathways on ESCs remains unclear.

Recently, several microRNAs(miRNAs or miRs) have been proved to play pivotal roles in skin development and pathogenesis, such as miR-203, miRNA21, miRNA146a/b, and miRNA34 [13, 14]. Chief among them is miR-203, a skin-specific miRNA, which is further corroborated as a key role in the regulation between proliferation and differentiation of ESCs [15, 16]. The ESCs lost their proliferative potential and embarked on a terminal differentiation program in light of the upregulation of miR-203. On the other hand, when miR-203 is downregulated, the ESCs populated suprabasal cell layers and differentiated into keratinocytes [17]. Given that miR-203 expression level is closely related to proliferative potential of ESCs, we hypothesized that miR-203 could play a central role in diabetic wound healing. Previously, we found that the expression of miR-203 was markedly upregulated in wounded skin of DM and the profile was positively correlated with the severity of diabetic foot ulcers [18]. However, little is known about how miR-203 acts on the proliferation of ESCs during the process of chronic wound healing in DM.

With the wound models constructed on DM rats in vivo and the isolated ESCs in vitro, we explored the role of miR-203 during the DM wound healing process, including the characteristics of its expression, alterations of ESCs proliferation and differentiation, the related signaling pathways, and putative target genes.

So far, the mechanism of DM wound healing is not fully understood, and there is no clinically effective treatment. The treatment of DM difficult wounds has become a very difficult problem in clinical practice. With the deepening of research, the healing effect of ESCs on DM wounds has gradually attracted researcher’s attention. In general, the more ESCs remaining on the wound, the faster the healing rate. Recent studies have shown that the proliferation and differentiation of ESCs in DM refractory wounds is dysfunctional, mainly due to decreased proliferative capacity, decreased number, decreased adhesion activity, and distribution changes of stem cells. ESCs’ proliferation and differentiation have been shown to be regulated by precise and orderly expression of many genes in time and space. Skin-specific expression of miR-203 is closely related to changes in the proliferation and differentiation of ESCs. The study is to systematically investigate spatial and temporal changes of miR-203 expression, epigenetic modification, the relationship of the related gene expression, and pathways during the process of DM wound healing in vivo DM wounds and in vitro ESCs model.

## Methods

### STZ induced DM rat model and established a rat wound model

Twenty-four SD rats were randomly divided into two groups; one group was a normal group of rats (12), and the other group was a DB rat (12); rats were fasted for 12–24 h before STZ injection. The longer the fasting time, the more obvious the destructive effect of STZ on insulin. Equipped with 1–2% concentration of citrate buffer, used within 30 min. STZ was dissolved in citrate buffer. The rats were injected intraperitoneally at a dose of 40 mg/kg. At 24 h and 48 h, the fasting tail vein blood glucose was higher than 16.7 mmol/L, and there were polydipsia, polyuria, polyphagia, and weight loss, which was regarded as a successful model. Continue to check blood glucose levels weekly. The unmolded mice can be remodeled after returning to normal state. The above rats were selected and anesthetized with 3% pentobarbital sodium (25 mg/kg) by intraperitoneal injection. After success, the back was depilated and disinfected. Under sterile conditions, a circular wound with a diameter of 2.8 cm and an area of 6.15 cm2 was made at the same position on the back of the mouse. The cut skin included epidermis, dermis, and subcutaneous fat. After waking, the rats were reared in cages, drinking water and feeding in a fixed amount, paying attention to the skin care of the rats to avoid wound infection. All experiments were completed in a clean animal laboratory.

### Isolation and culture of ESCs

Pregnant Sprague–Dawley (SD) rats were obtained from the Experimental Animal Center of Sun Yat-Sen University and were kept under standard conditions according to the ethical committee of the Medical Sciences Department. In this study, we used fetal rats (19 days to 21 days gestational age). After the sacrifice of rats, the skin from the torso of each rat was harvested, rinsed twice with D-Hanks buffer, and immersed in D-Hanks buffer containing 200 U/mL penicillin and 200 U/mL streptomycin (Hyclone, Cat. No. SH30010) for 30 min. Under sterile conditions, the skin was washed thoroughly with PBS to remove the subcutaneous tissue and trimmed into 0.5 × 0.5 cm pieces. The skin was digested at 4 °C overnight in digestion buffer containing 0.5% neutral protease (GIBCO Cat. No. 17105041). In the following morning, the skin samples were incubated at 37 °C for 30 min. After peeling off the epidermis and cut into the microskin, the skin sample was oscillated and digested with 0.25% trypsin (Hyclone, Cat. No. SH3008742.01) at 37 °C for 15 min until a single-cell suspension was formed. The digestive reaction was stopped by the addition of high-glucose Dulbecco’s modified Eagle’s medium (DMEM) containing 20% fetal bovine serum (FBS) (Hyclone, Cat. No. SH30022.01B) in accordance with the volume of trypsin. Cells were filtered with a 200-mesh filter and centrifuged at 1000 rpm for 5 min. After the removal of the supernatant, the cells were resuspended in a high-glucose DMEM containing 20% FBS and seeded at a cell density of 3 × 10^6^/mL in a flask coated with type IV collagen (Sigma Cat. No. C8374). After 15-min incubation in 37 °C, the cells were observed under an inverted phase contrast microscope, and adhesion of cells to the bottom of the flask would be monitored. The suspended cells were then collected, and 4 mL of high-glucose DMEM containing 20% FBS (Hyclone, Cat. No. SH30022.01B) was added to the adherent cells and cultured in a saturated humidified atmosphere of 5% CO_2_ at 37 °C. The culture medium was exchanged to K-SFM medium (GIBCO, Cat.No.17005042) after 24 h, and the cells were passaged regularly. Half of the medium was replaced every other day, and the complete medium was replaced every 2 to 3 days. When the culture reached a confluence of 70 to 80%, the cells were digested in 0.25% trypsin at 37 °C with a 5- to 10-min oscillation and passaged at a ratio of 1:2.

### Immunocytochemistry

Cells were fixed with 4% paraformaldehyde for 30 min, washed with PBS thrice for 5 min, and incubated with 3% peroxide in a humidity box at room temperature for 10 min. After three PBS washes for 5 min each, the cells were blocked with 10% normal goat serum at room temperature for 30 min. Incubation with primary antibodies was performed at 4 °C overnight. The primary antibodies used were rabbit anti-CK15 (BIOSS Cat. No. bs-1772R), rabbit anti-CK19 (BIOSS Cat. No. bs-1028R), and rabbit anti-P63 (BIOSS Cat. No. bs-0723R). Following 3 × 5 min washes with PBS, cells were incubated with the secondary antibody at room temperature for 30 min, washed with PBS thrice for 5 min each, and incubated with DAB using ChemMate TM Dako Envision TM Detection Kit (Dako, GK500705). Staining was stopped by washing with PBS thrice. Cells were counterstained with hematoxylin. The superfluous staining was removed with water. Cells were dehydrated with 50%, 75%, 85%, 95%, and 100% gradients of ethanol (once per step) and cleared with xylene twice for 10 min prior to mounting in neutral resin. An Olympus CX41 microscope was used to observe cells.

### Immunohistochemistry and immunohistofluorescence assays on tissue samples

Skin samples were fixed in 4% formalin solution, embedded in paraffin, and sectioned (5 μm) for immunohistochemistry and immunohistofluorescence. Immunohistochemistry staining was used to detect expression of Notch1. Paraffin sections were subjected to antigen retrieval using a pressure cooker, in sodium citrate (pH 6.0), for 4 min. Endogenous peroxidase was blocked with 3% hydrogen peroxide (H_2_O_2_) in PBS followed by nonspecific blocking with 2% PBS + bovine serum albumin (BSA) for 15 min. The sections were incubated with the primary antibody overnight at 4 °C. The chromogenic reagent DAB was used to show the antibody conjugation. The intensity of the reaction observed on the slides was qualitatively analyzed.

Double-immunolabeling was used to detect Hes1 and BrdU in epidermis during wound healing. Formalin-fixed sections were deparaffinized in xylene and rehydrated in graded alcohols. Tissue sections were microwaved in 10 mM sodium citrate (pH 6.0) for 3 min, incubated for another 15 min in the hot solution, and rinsed in Automation Buffer (Biomedia, Foster City, CA). Sections were incubated in 2 M HCl at 37 °C, washed in borate buffer, and digested in 0.01% trypsin in 0.05 M Tris for 3 min at 37 °C. After blocking in 10% goat serum for 20 min, sections were incubated for 1 h at room temperature with mouse BrdU anti-sera(Becton Dickinson; 1:25) and Hes1 anti-sera (1:100) in 1% bovine serum albumin.

### Cell proliferation assay

Cells from different groups were digested, dispersed by pipetting, and then counted. The cell concentration was adjusted to 1 × 10^5^ cells/mL, and cells were distributed in a 96-well microplate (100 μL/well, i.e., 1 × 10^4^ cells/well). After cell adhesion, the cells were collected at different time points (0 and 72 h). MTS was added at a ratio of 1/10 (i.e., 10 μL of detection solution was added to 100 μL of medium) according to the instructions in the CellTiter96® Aqueous One Solution Cell Proliferation Assay (MTS) (Promega, Cat. No. G3582). After incubation for 4 h, the MTS levels were read at OD 490 using a microplate reader (Thermo Fisher Scientific, Multiscan MK3).

### MTT assay

Cell growth was measured by MTT assay. MTT cell proliferation kits were purchased from BioVision Technologies (Exton, PA, USA). Cell proliferation was measured according to the manufacturer’s protocol. The cells were portioned in a 96-well plate at 2000 cells/well to 5000 cells/well. Cells were cultured in DMEM, either with or without FBS, depending on the individual experiments. Samples were assayed in triplicates and experiments were repeated thrice.

### Histological and immunohistochemical studies on HSCs

Standard HE staining and dual-color immunofluorescence techniques were used throughout the study. For each antibody, staining was performed on at least three mice of each genotype, and the average staining intensity over the entire tissue area was scored. Representative images were obtained for each staining. Isotype-matched control antibodies (eBiosciences) were used as a negative control. For semi-quantification, positive signals in at least five random high-power fields were visualized, counted, and expressed as the percentage of total DAPI-positive cells.

### Western blot analysis

Cell lysates or rodents’ skin homogenates (50 to 100 μg of total protein) were separated on a polyacrylamide–sodium dodecyl sulfate gel and electro-blotted onto a nitrocellulose membrane (BioRad, Hercules, CA, USA). The proteins were incubated overnight with the antibodies, transferred to a PVDF membrane (Millipore, MA, USA), and detected for protein expression using an enhanced chemiluminescence (ECL; ECL Western Blot Substrate, Pierce, USA) system.

### RNA extraction and real-time PCR analysis

RNA was extracted using a single-step method of TRIzol (Invitrogen). RNA concentration and purity were measured using a Nanodrop spectrophotometer, and cDNA was synthesized from 1 μg of total RNA using RevertAid H minus first-strand cDNA synthesis kit (Fermentas) according to the manufacturer’s instructions. Quantitative real-time PCR was performed with a 7500 Real-Time system using Fast SYBR Green Master Mix (Applied Biosystems), and the primers are listed in Table 1. After normalization to GAPDH mRNA, relative expression levels and fold induction of each target gene were calculated by comparative CT method [(1/2) formula, where ΔCT is the difference between CT–target and CT–reference] using Microsoft Excel 2007.

**Table 1:**
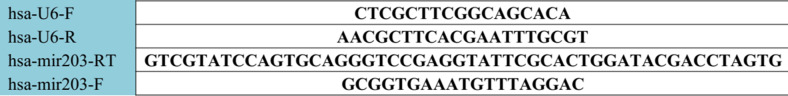
Primer sequence

### Statistical analysis

All values are expressed as mean ± SD. Student’s paired *t* test was performed for comparison of data from paired samples. Analysis of variance was used for comparisons among multiple groups, followed by Friedman’s post hoc test. A probability value *p* < 0.05 was considered significant.

## Results

### Expression levels of miR-203 was elevated in DM rats

**Fig. 1:**
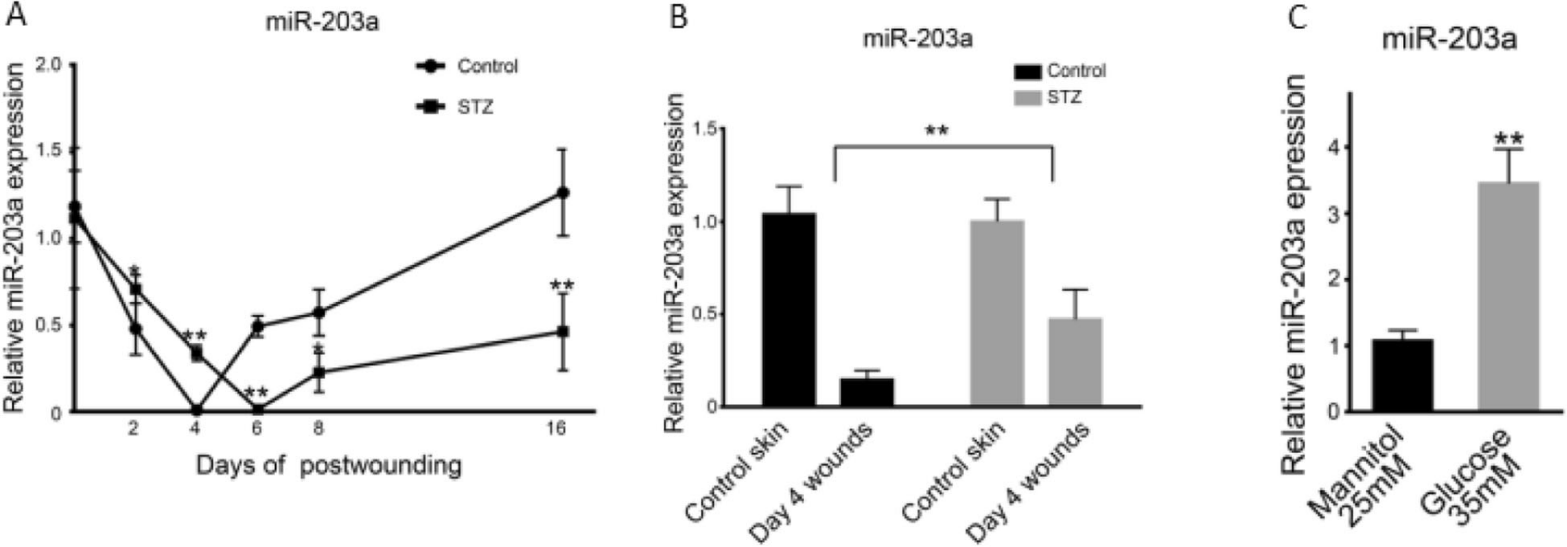
Expression level of miR-203. Expression level of miR-203 during wound healing process in control and STZ group (**a**). Comparisons of miR-203 expressions between control and STZ group (**b**). Expression level of miR-203 in (35 mmol/l glucose) and mannitol (25 mmol/l)-incubated ESCs (**c**). Data are representative profile of clustered miRNA in individual samples and expressed as the mean ± SD of each group of samples from at least three separate experiments. *p < 0.05; **p < 0.01; ***p < 0.001

We first assessed the expression levels of skin-specific miR-203 in wound models of normal and STZ-treated (type 2 diabetes) rats. In normal rats, the miR-203 expression level declined during the early phage of wound healing and bottomed out by day 4, but then returned to normal levels. In rats with type 2 diabetes, the level declined more slowly and reached the trough level by day 6, then showed delayed recover. (Fig. 1a). After 4 days in the DM model, the miR-203 expression level of wounded skin decreased. In vitro, the ESCs were exposed to high glucose medium and the miR-203 expression was assessed [19] (Fig. 1b). Compared with (isosmotic) mannitol treatment group, the ESCs exhibited a higher expression level of miR-203 under high glucose treatment (Fig. 1c). These findings suggested that the expression level of miR-203 is elevated during the healing process of chronic wounds in DM and it is caused by high glucose.

### Over-expression of miR-203 reduced the number and proliferation capacity of ESCs

**Fig. 2:**
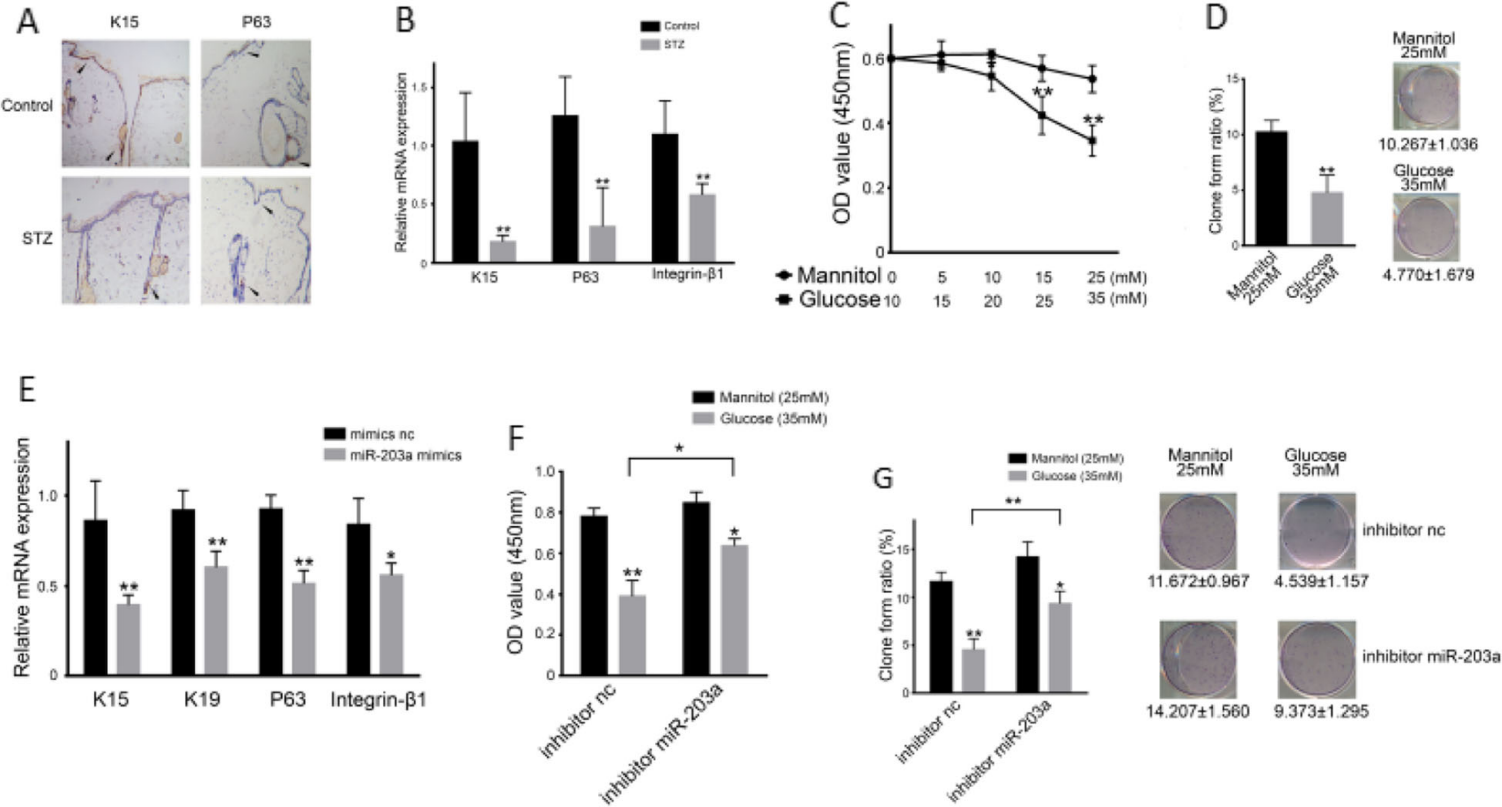
Overexpression of miR-203 reduce the number of ESCs and impaired their proliferation capacity. K15+ and P63+ cell in normal rats and rats with type 2 diabetes wounded skin (**a**). mRNA expression of K15, P63, and Intergrin-β1 in normal rats and rats with type 2 diabetes wounded skin (**b**). Colony-forming assay for mannitol/glucose-incubated ESCs (**c**, **d**). Overexpression of miR-203reduce expression of K15, K19, P63, and Intergrin-β1 (**e**). Inhibition of miR-203 improve colony-forming assay for mannitol/glucose-incubated ESCs (**f**, **g**). Data are representative profile of clustered miRNA in individual samples and expressed as the mean ± SD of each group of samples from at least three separate experiments. *p < 0.05; **p < 0.01; ***p < 0.001

We then assessed the expression of K15 and P63, known as the surface markers of ECSs, by IHC and RT-PCR. K15 and P63 IHC expression (Fig. 2a) and mRNA levels (Fig. 2b) were reduced drastically in wounded skin of DM rats. As depicted in Fig. 2b, the mRNA level of integrin-β1 was also declined, suggesting that ESCs were greatly reduced in DM rats’ skin tissues. Consistent with these observations in rats, in vitro simulation revealed that high glucose treatment lowered the OD value of MTT, representing the number of ESCs, in a dose-dependent manner (Fig. 2c). The ESCs’ proliferation was also decreased (Fig. 2d). We then conducted experiments to overexpress as well as knock down the miR-203 in ESCs. K15, P63, and intergrin-β1 decreased significantly with over-expression of miR-203 (Fig. 2e). However, knocking down miR-203 reduced the loss of ESCs caused by high glucose treatment (Fig. 2f), suggesting that miR-203 plays a role in high-glucose-induced loss of ESCs (Fig. 2g). Therefore, the overexpression of miR-203 results in reduced quantity and limited proliferation of ESCs.

### Overexpressed miR-203 decreased the proliferation of ESCs by inhibiting the Wnt and Notch signaling pathways

**Fig. 3:**
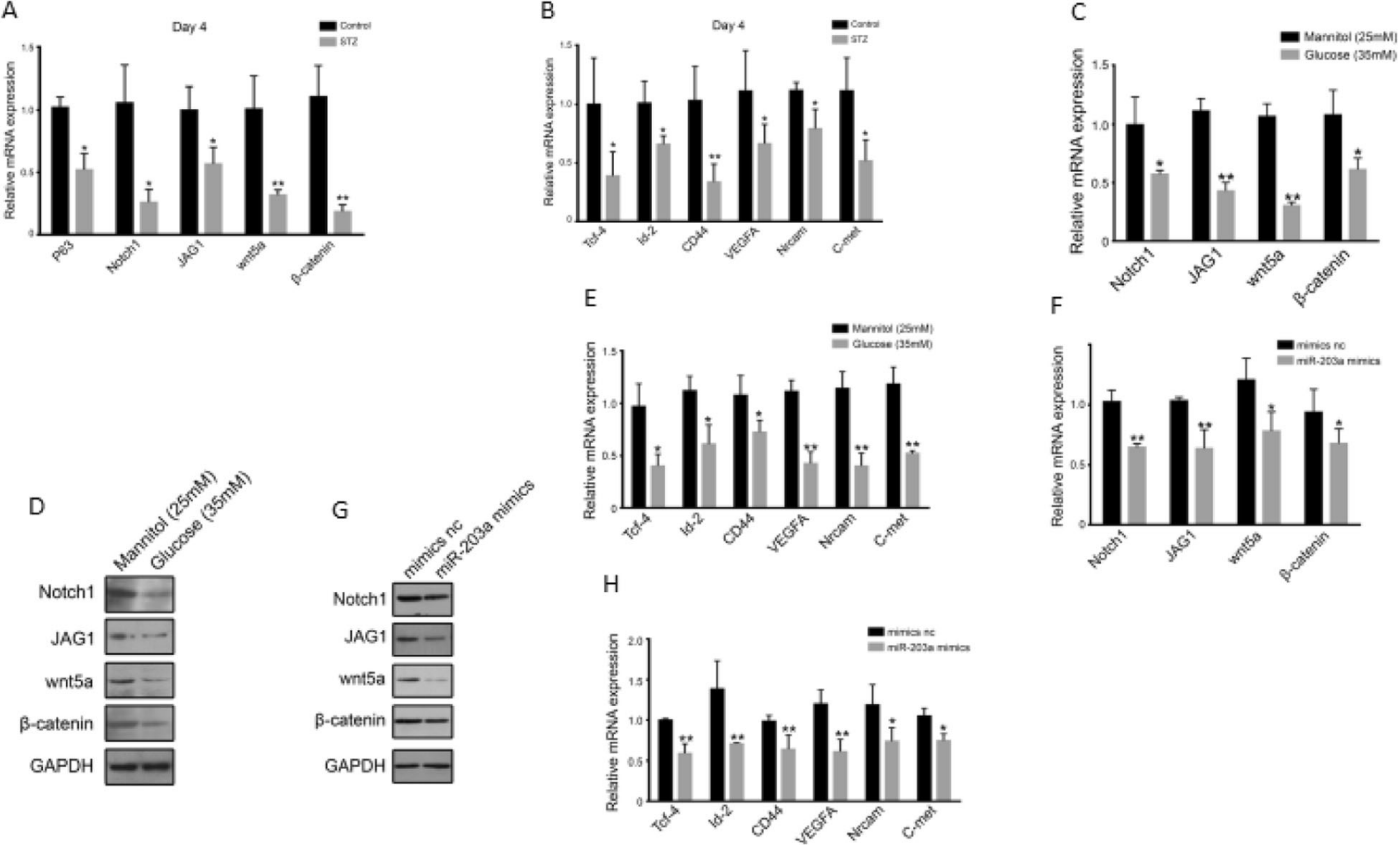
miR-203’s depression on Wnt and Notch causes weak ESCs proliferation. Expression of Wnt and Notch signaling pathway in rat with diabetes mellitus by qRT-PCR (**a**). Expressions of Wnt’s and Notch’s downstream in rat with diabetes mellitus by qRT-PCR (**b**). Expression of Wnt and Notch signaling pathway in ESCs co-cultured with high glucose by qRT-PCR (**c**). Expression of Wnt and Notch signaling pathway in ESCs co-cultured with high glucose by WB (**d**). Expression of Wnt’s and Notch’s downstream in ESCs co-cultured with high glucose by qRT-PCR (**e**). Expression of Wnt and Notch signaling pathway in miR-203a-overexpressed ESCs by qRT-PCR (**f**). Expression of Wnt and Notch signaling pathway in miR-203a-overexpressed ESCs by WB (**g**). Expressions of Wnt’s and Notch’s downstream in miR-203a-overexpressed ESCs by qRT-PCR (**h**). Data are representative profile of clustered miRNA in individual samples and expressed as the mean ± SD of each group of samples from at least three separate experiments. *p < 0.05; **p < 0.01; ***p < 0.001

The Wnt and Notch signaling pathways are important players in controlling the ESCs’ signaling microenvironment stem cell niches [5, 6]. IHC and RT-PCR were performed on Wnt, Notch, and downstream genes including TCF-4, ID-2, CD44, VEGFA, NRCAM, and C-MET in DM and normal rats’ wounded skin tissues. At day 4 in high glucose group, mRNA expression of Wnt and Notch were decreased significantly, while downstream regulatory genes were downregulated obviously (Fig. 3a, b). In HaCat cells, high glucose treatment for 96 h lowered the mRNA and protein level of Notch and Wnt (Fig. 3c, d). Downstream regulatory genes also declined in the presence of high glucose (Fig. 3e). Similarly, a miR-203 mimic, after transfected into ESCs, also significantly downregulated the expression of Notch, Wnt-related molecules (Fig. 3f, g), and the expression of downstream regulatory genes (Fig. 3h). These results suggested that high expression of miR-203 in DM rats’ wounded skin tissues decreased the expression of Notch, Wnt, and their downstream regulatory genes and weakened and reduced ESCs’ proliferation, which may contribute to the slow recovery of chronic wounds in DM.

### Suppression of miR-203 reversed the changes of ESCs’ functions after high-glucose treatment and promoted the healing of chronic wounds in DM

**Fig. 4:**
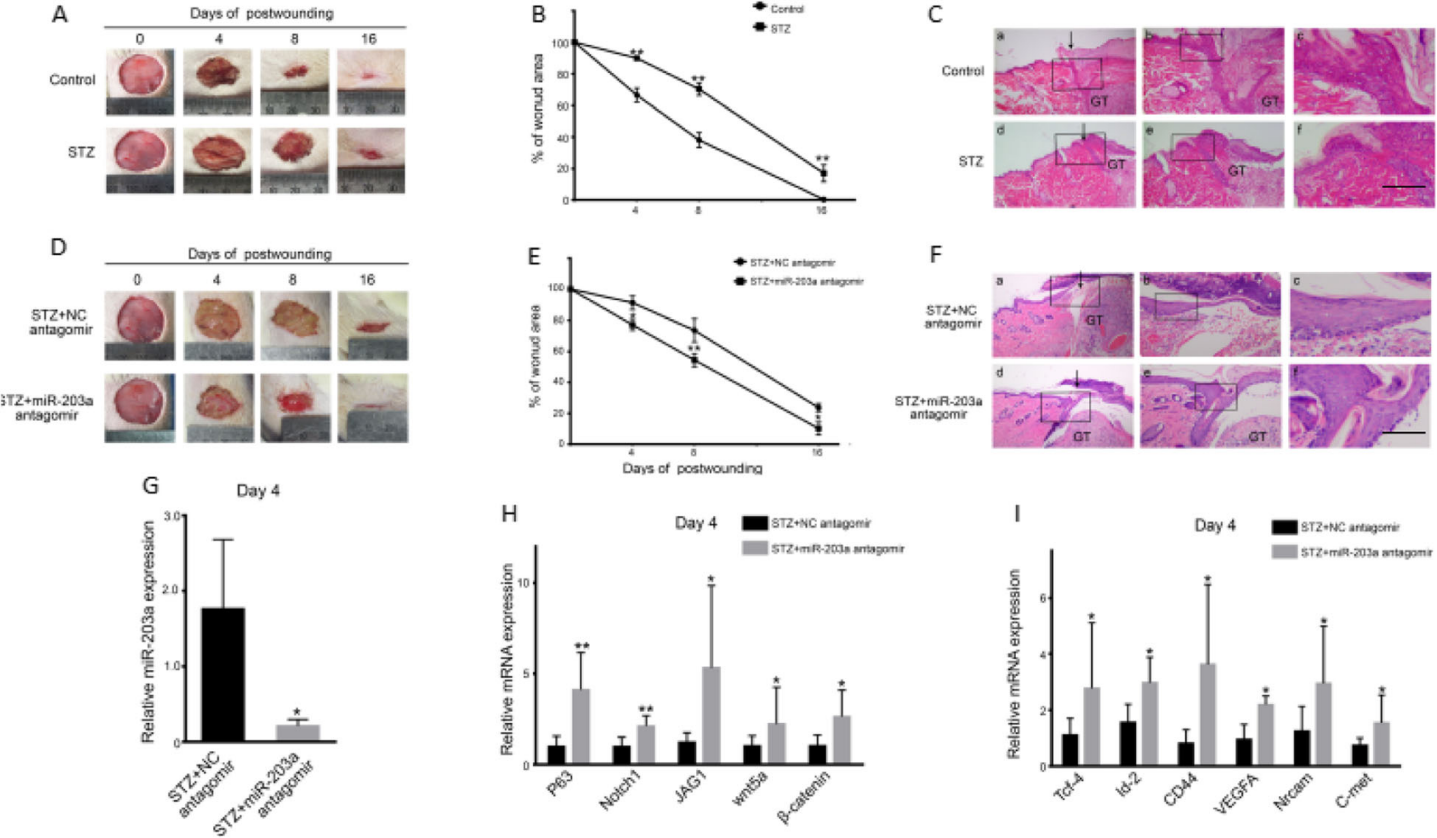
miR-203 depression can do good to repair damage to ESCs. Speed and quality of wound healing in rats with diabetes mellitus (**a**–**c**). Speed and quality of wound healing in rats with diabetes mellitus and overexpressed miR-203a (**d**–**g**). Expression of Wnt and Notch signaling pathway in rats with diabetes mellitus and overexpressed miR-203a (**h**). Expression of Wnt’s and Notch’s downstream in rats with diabetes mellitus and overexpressed miR-203a (**i**). Data are representative profile of clustered miRNA in individual samples and expressed as the mean ± SD of each group of samples from at least three separate experiments. *p < 0.05; **p < 0.01; ***p < 0.001. Scale bar, 50 μm

A delayed recovery of wounds was observed in DM rats, comparing to normal rats (Fig. 4a, b). HE staining revealed that, in day 4, skin tissues in DM rats were thinner and exhibited abnormal skin structure. We observed shrinkage and degradation of dermal collagen, indicating that the healing response in the skin of DM rats was impaired (Fig. 4c). To further demonstrate the effect of miR-203 on wound healing in DM rats, a miR-203 antagomir was used through multi-point subcutaneous injection. The results revealed that the healing time was shortened greatly (Fig. 4d, e). We then used an antagomir to miR-203, which robustly decreased miR-203 by day 4. (Fig. 4f). The antagomir modified skin structure through thickening the skin and restoring dermal collagen (Fig. 4g). The miR-203 antagomir also restored the expression of Notch and Wnt-related molecules once reduced by miR-203 (Fig. 4h, i).

### ROCK2, MAPK8, MAPK9, and PRKCA were the potential downstream targets of the Wnt/β-catenin signaling pathway after the alteration of miR-203

**Fig. 5:**
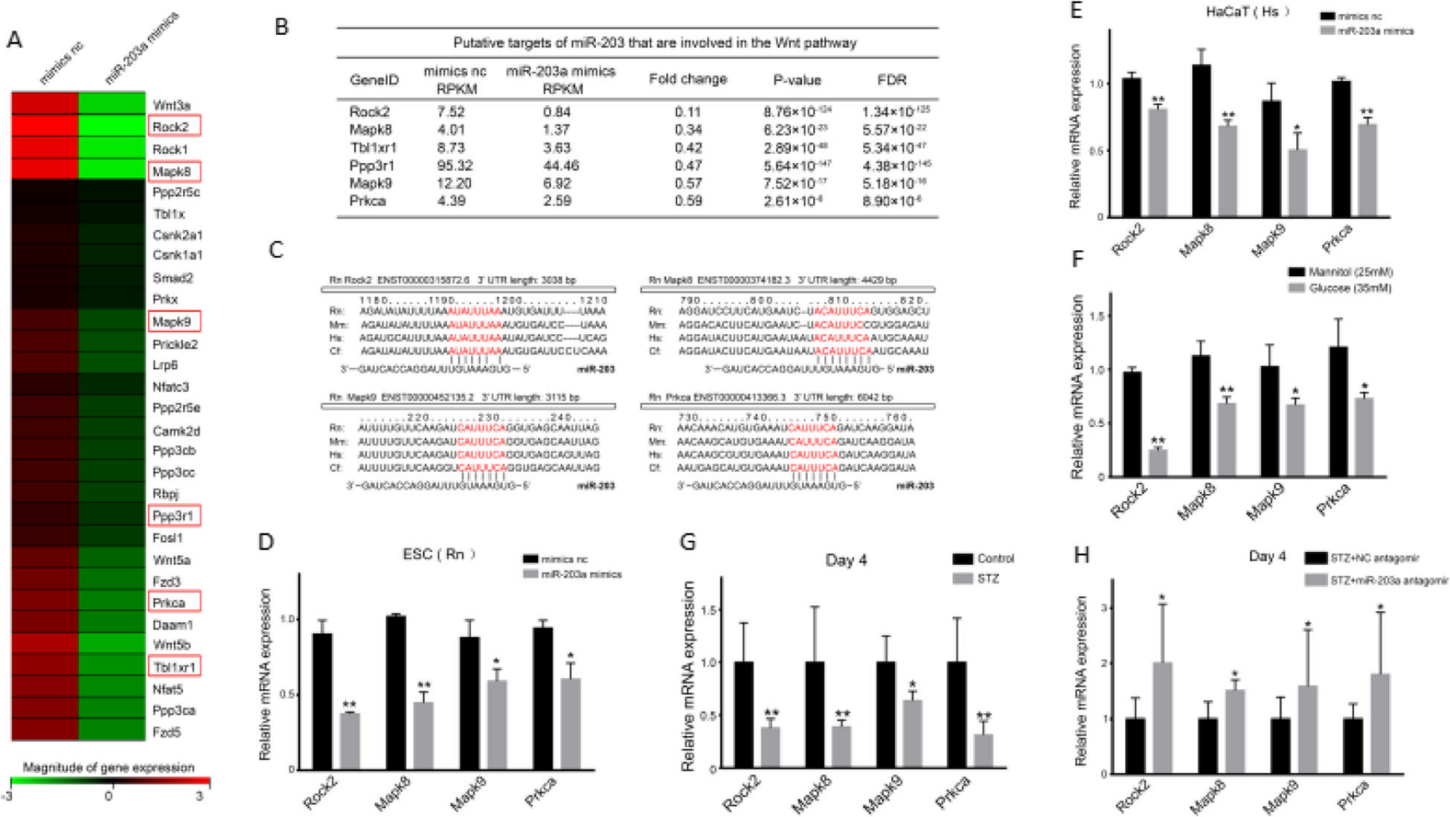
Potential target genes of miR-203 are ROCK2, MAPK8, MAPK9, and PRKCA. Gene expression pattern in rats with diabetes mellitus and overexpressed miR-203a by gene sequencer (**a**, **b**). Target gene expression in miR-203a-overexpressed ESCs from Rn, miR-203a-overexpressed HaCaT, and miR-203a-overexpressed ESCs from rats (**c**–**e**). Target gene expression in rats with diabetes mellitus (**f**). Target gene expression in rats with diabetes mellitus and overexpressed miR-203a (**g**). Data are representative profile of clustered miRNA in individual samples and expressed as the mean ± SD of each group of samples from at least three separate experiments. *p < 0.05; **p < 0.01; ***p < 0.001

To investigate potential target genes, cells were transfected with NC and miR-203, then analyzed by gene sequencing, which led us to identify downregulated genes such as ROCK2, MAPK8, MAPK9, PRKCA, Tbl1xr1, and Ppp3r1 (Fig. 5a, b). Intriguingly, ROCK2, MAPK8, MAPK9, and PRKCA (Fig. 5c) had complementary base pairs to miR-203. Furthermore, overexpression of miR-203 decreased the expression of ROCK2, MAPK8, MAPK9, and PRKCA (Fig. 5d), which we also found in HaCat cells (Fig. 5e). The expression of ROCK2, MAPK8, MAPK9, and PRKCA was also declined in high glucose group but not mannitol control (Fig. 5f). Moreover, in DM rats by day 4, the expression of ROCK2, MAPK8, MAPK9, and PRKCA was decreased in wounded skin tissues (Fig. 5g) and increased after the utilization of miR-203 antagomir (Fig. 5h). These studies suggested that miR-203 may participate in wound healing process through ROCK2, MAPK8, MAPK9, and PRKCA.

## Discussion

Our studies revealed that (1) miR-203 expression was related to the healing process of chronic wounds in DM; (2) a high expression level of miR-203 in DM wound was associated with glucose exposure, which in turn inhibited the Notch and Wnt signaling pathways, and further suppressed their downstream regulatory genes, and consequently led to reduction of ESCs proliferative capacity. We believe that these defects of signaling pathways were of paramount importance in the impaired healing process of chronic wounds in DM; (3) furthermore, we found that utilizing a miR-203 antagomir ameliorated the defects on the healing process in DM wounds; (4) ROCK2, MAPK8, MAPK9, and PRKCA might be new potential target genes of miR-203 when regulating wound healing process via the Notch and Wnt signaling pathways.

It has been shown that miR-203 plays a pivotal role in the development, formation, and function of skin tissues [20–22]. Intentionally removing Dicer or dgcr8 (key enzymes in biological formation of miRNA) in horn cells of rats led to reduced skin ESCs, shielding dysfunction, hair dysplasia, and over-proliferation of horn cells among basal follicles [23, 24]. MiR-203, as a specific expressed miRNA, could therefore play novel roles in the development, formation, and function of skin [20–22, 24]. Previous studies reported that in the early stage of skin development, the monolayer epidermal progenitor cells did not express miR-203 until embryonic phase in 13.5 days [17, 25]. However, during the differentiation of skin, the expression level of miR-203 was elevated. Our data suggested that when miR-203 is suppressed or lost, potential proliferative cells will expand beyond basal layer which may potentiate differentiation into horn cells, indicating that miR-203 is critical for skin development through regulating differentiation of ESCs. Other studies have shown that miR-203 expression level decreased in wound rim during wound healing [26]. We found that the expression of miRNA in dermal wounds reduced greatly and the keloid showed significantly lower miRNA expression relative to normal skin tissue [27]. Some studies showed that transfected miR-203 could regulate the expression of P63 as well as the proliferation and differentiation of ESCs, suggesting that miR-203 plays an important role in skin re-epithelialization and homeostatic reconstruction [16, 28]. In human, the miR-203 expression in DM wounds was upregulated versus normal wounds, and the specific quantity is correlated with the severity of wound [18]. In our experiments, after wound formation, normal rats showed decreased expression level of miR-203 but returned to normal levels soon thereafter, which indicates that the ESCs of normal rat wounds mainly reproduced in early time and differentiated in advance to heal. In contrast, the expression of miR-203 in DM rats declined and recovered slower, leading to a higher expression level of miR-203 in early stage and lower expression in advanced stage. This temporal pattern indicated that the proliferation of ESCs in the wounded areas of DM rats was reduced in early stage of injury and the differentiative capability was reduced in later stages. This pattern was closely related to chronic wounds in DM.

Wnt and Notch signaling pathways are important participants in maintaining ESCs’ microenvironment in stem cell niches [10, 11]. Studies have shown that the activation of Wnt signaling pathway could induce stem cells differentiate into hair follicle and sebaceous glands, while blocking Wnt signaling pathway led to differentiation of epidermal stem cells into epidermis [29, 30]. The Notch signaling pathway had a decisive regulatory effect on ESCs’ self-replication and differentiation. Stem cells would proliferate if Notch receptor is activated by its ligand; oppositely, suppressed Notch signaling pathway would convert them into functional cells [31–33]. Previous studies have shown that Wnt and Notch signaling pathways are deemed to be highly related to wound healing. Healing of chronic wound might have signaling dysfunctions, but the molecular mechanisms of how the Wnt and Notch signaling pathways are regulated in DM wounds remain unclear. According to recent researches, the ESCs contribute to wound healing process in DM via the Notch signaling pathway [34]. Our results here in this paper demonstrated that during healing process of DM wounds, the expression level of both Wnt and Notch signaling pathways were reduced. Meanwhile, over-expression of miR-203 would lead to suppression of Wnt and Notch signaling pathways. So, the Wnt and Notch signaling pathways would be vital in the regulation of miR-203 in wound healing in DM.

A certain kind of miRNA is capable of regulating several genes, while a gene can be regulated by multiple miRNAs [35]. According to previous studies, miR-203 has multiple targets. MiR-203 is a key factor in psoriasis since it could regulate SOCS3 and lead to the activation of STAT3 [36, 37], hence controlling the proliferation and differentiation of ESCs in skin development and wound healing through P63 and Zfp281 [38]. Our experiment utilized cDNA microarray technology to test different ESCs’ miRNA expression profile, which showed that the expression levels of ROCK2, MAPK8, MAPK9, and PRKCA could be reduced greatly. In addition, pair analysis showed that ROCK2, MAPK, MAPK9, and PRKCA have complementary base pairs with miR-203. Therefore, it would be convincing that ROCK2, MAPK8, MAPK9, and PRKCA could be potential new targets, and according to bioinformatic analysis, ROCK2, MAPK8, MAPK9, and PRKCA are closely related to the Wnt signaling pathway.

## Conclusion

In summary, the study data suggest that induced expression of miR-203 by high glucose in type 2 diabetic rats might decrease the number of ESCs and impaired ESC proliferation capacity via downregulating genes related to Notch and Wnt signaling pathways, resulting in a delayed wound healing. And furthermore, we identified that ROCK2, MAPK8, MAPK9, and PRKCA, whose proteins related to Wnt/β-catenin pathway, are novel target genes of miR-203.

## Data Availability

The data supporting the conclusions of this article are included within the article.

## Abbreviations

ESCs: Epidermal stem cells
miR-203: miR-203
miRNAs: MicroRNAs
DM: Diabetes mellitus
AGEs: Advanced glycation end-products
MMPs: Matrix metalloproteinases
SD: Sprague–Dawley
DMEM: Dulbecco’s modified Eagle’s medium
FBS: Fetal bovine serum
H2O2: Hydrogen peroxide
BSA: Bovine serum albumin
